# Notes and Comments

**Published:** 1899-05

**Authors:** 


### Notes and Comments.[1]

A CHINESE NAIL.—Isaac T. Headland, professor in Peking University, tells in *The Advance* of a man who came to receive treatment in a hospital in China, who had given many years of his life to nail-culture. From his seventeenth year he had allowed

[1] The assistant editor solicits contributions for this department,—new methods, new remedies and formulas, or any short practical note which may prove of value to the practitioner or student. Address 1338 Walnut Street Philadelphia.

the nails on his third and little fingers to grow without trimming, and when Dr. Curtiss measured them he was in his fortieth year. The nails were one foot in length from the ends of the fingers. The man "had fitted small bamboo tubes on the ends of his fingers as shields for his nails, and thus had protected them for twenty-three years." He seemed to think that he had not lived in vain, but absolutely he had done nothing notable except to make his hands useless by developing two nails.

---

REPAIRING GOLD CROWNS.—Dr. E. A. Randall, in the *Dominion Dental Journal,* gives the following method of repairing gold crowns. Suppose you have made a gold crown, and in finishing you go through the shell, making an unsightly hole. If you undertake to solder this, the chances are that you will have three or four holes caused by the solder melting out at the joints. To prevent this trouble, paint the crown all over the outside with whiting mixed thin, except around the hole which you wish to repair; fill this with a plug made from gold-foil, touch it up with a drop of borax water, and put a bit of gold solder inside, heat it with the blow-pipe, and success will be the result.

---

COLOR IN IMPLANTATION.—In all natural inlays or implanted teeth the part inserted will take on the color of the adjoining teeth or the adjoining part. That is a peculiarity in implanted teeth; but you must use a tooth, or section of tooth, of the same temperament as that upon which you are working. For instance, if a tooth is of a bilious temperament, or a nervous temperament, you want to use one of that temperament, not take the tooth of a bilious temperament for an operation upon the tooth of a nervous temperament. The teeth of the nervous temperament are of a bluish color; the bilious temperament of a yellowish color. With that care you can match a tooth almost perfectly.—*Dental Brief.*

---

THE DISPLAY OF GOLD IN TEETH.—Professor E. T. Darby, in writing upon the subject, says, "I am frequently shocked, when I see the mouths of some people, to find glaring gold crowns on the bicuspids and anterior teeth. Only last week I met a lady in a trolley car in Philadelphia whom I had known years ago, but who for some years past has been living in one of the Southern States. As soon as I had entered into conversation with her I observed that she had three gold crowns on her upper bicuspids, and a great display of gold in the incisors and bicuspids. A pretty face almost ruined by this shocking display of gold.

"I have sometimes thought that this craze for crowning teeth with gold was more prevalent in the Western and Southern States than in the Middle and Eastern States, but it may be that my attention has been more frequently called to cases coming from those sections of the country."

